# Germination as a Sustainable Green Pre-Treatment for the Recovery and Enhancement of High-Value Compounds in Broccoli and Kale

**DOI:** 10.3390/molecules31020350

**Published:** 2026-01-19

**Authors:** Christine (Neagu) Dragomir, Corina Dana Misca, Sylvestre Dossa, Daniela Stoin, Ariana Velciov, Călin Jianu, Isidora Radulov, Mariana Suba, Catalin Ianasi, Ersilia Alexa

**Affiliations:** 1 Faculty of Food Engineering, University of Life Sciences “King Mihai I” from Timisoara, Aradului Street No. 119, 300645 Timisoara, Romania; christine.neagu@usvt.ro (C.D.); dossasylvestre@usvt.ro (S.D.); danielastoin@usvt.ro (D.S.); arianavelciov@usvt.ro (A.V.); calinjianu@usvt.ro (C.J.); 2 “Food Science” Research Center, University of Life Sciences “King Mihai I” from Timisoara, Aradului Street No. 119, 300645 Timisoara, Romania; 3 Faculty of Agriculture, University of Life Sciences “King Mihai I” from Timisoara, Aradului Street No. 119, 300645 Timisoara, Romania; isidora_radulov@usvt.ro; 4 “Coriolan Dragulescu” Institute of Chemistry, Romanian Academy, Mihai Viteazu No. 24, 300223 Timișoara, Romania; marianasuba@gmail.com (M.S.); ianasic@acad-icht.tm.edu.ro (C.I.)

**Keywords:** *Brassicaceae*, germination, proximate composition, macro and microelements, FTIR, SAXS/WAXS, antimicrobial activity

## Abstract

Germination is widely recognized as an effective strategy to enhance the nutritional quality and reduce anti-nutritional factors in plant foods. This study evaluated the impact of germination on Cruciferous vegetables (family *Cruciferae* or *Brassicaceae*) broccoli and kale by assessing changes in proximate composition, macro- and microelement profiles, total and individual polyphenols, phytic acid content, antimicrobial activity, and structural characteristics using Fourier Transform Infrared Spectroscopy (FTIR) and Small- and Wide-Angle X-ray Scattering (SAXS/WAXS) analyses. Germination significantly increased protein content (30.33% in broccoli sprouts and 30.21% in kale sprouts), total phenolic content (424.40 mg/100 g in broccoli sprouts and 497.94 mg/100 g in kale sprouts), and essential minerals, while reducing phytic acid levels in both species (up to 82.20%). Antimicrobial effects were matrix-dependent, being detected in broccoli and kale seed powders, while no inhibitory activity was observed for the corresponding sprout powders under the tested conditions. FTIR spectra indicated notable modifications in functional groups related to carbohydrates, proteins, and phenolic compounds, while SAXS analysis revealed structural reorganizations at the nanoscale. Overall, germination improved the nutritional and phytochemical quality of broccoli and kale while decreasing anti-nutritional compounds, highlighting its potential to enhance the health-promoting value of *Brassica* sprouts.

## 1. Introduction

Cruciferous vegetables (family *Cruciferae* or *Brassicaceae*)—including broccoli (*Brassica oleracea* var. *italica*) and kale (*Brassica oleracea* var. *acephala*)—represent one of the most important botanical groups recognized for their dense nutritional profile and rich content of bioactive phytochemicals. These vegetables have gained considerable attention as functional foods, due to their capacity to provide health benefits that go beyond basic nutrition [1].

Kale (*Brassica oleracea* var. *acephala*) is a cruciferous vegetable characterized by its non-heading leafy structure along the stem. In recent years, it has gained remarkable popularity as a so-called “superfood.” Consequently, it is frequently featured in popular culture and scientific literature among the “healthiest vegetables,” owing to its rich nutritional profile and diverse array of bioactive compounds [1]. Cultivated for more than two millennia, kale is currently widespread in Mediterranean and Asian regions. It is recognized as a valuable source of bioactive compounds, including flavonoids, polyphenols, carotenoids, glucosinolates, and dietary fibers, which contribute to its high nutritional and functional value [2].

Broccoli (*Brassica oleracea* var. *italica*) is a biennial cruciferous vegetable that exemplifies the growing need for a comprehensive food composition database. The global production and consumption of broccoli has increased dramatically in recent decades, reflecting a growing awareness of its rich nutritional profile and beneficial health properties [3,4,5].

In the context of the increasing interest in healthy eating and products with high functional value, sprouts and microgreens have attracted particular attention as concentrated sources of nutrients and bioactive compounds. They are obtained by germinating seeds under controlled temperature and humidity conditions and can be consumed as such or included in various food products. Recent studies highlight that sprouts and microgreens have high nutritional density, increased content of vitamins, minerals, and phenolic compounds, as well as significant antioxidant activity, being considered promising ingredients for healthy and sustainable diets [6,7]. Sprouts also appear to be an important source of essential minerals and trace elements that are easily absorbed by the human body [8]. Germination can be regarded not only as a growth stage but also as a biological pre-treatment that promotes the release and mobilization of bioactive compounds. The activation of endogenous hydrolytic enzymes during germination leads to partial degradation of the plant cell wall and storage macromolecules, facilitating the liberation and increased bioavailability of bound phenolics and nutrients. In this context, germination functions as a green bio-extraction strategy, enabling the recovery of high-value compounds without the need for harsh solvents or energy-intensive processing [9,10].

In recent years, numerous syntheses and experimental studies have been published confirming the role of sprouts as sources of bioactive compounds, including polyphenols, carotenoids, glucosinolates and bioactive peptides, with potentially beneficial impacts on cardiovascular, metabolic and digestive health [11,12]. A particularly intensively studied case is broccoli sprouts (*Brassica oleracea* var. *italica*), recognized for their extremely high content of glucoraphanin, a precursor of sulforaphane, an isothiocyanate with potent antioxidant and anti-inflammatory properties, being investigated for its role in modulating oxidative stress, chronic inflammation and the detoxification response mediated by the transcription factor [13,14].

Recent clinical data indicate that the intake of broccoli sprouts rich in sulforaphane can improve metabolic parameters, including glycemia and insulin resistance, in patients with type 2 diabetes or obesity, as well as markers of oxidative stress and inflammation [15,16]. The study performed by van Steenwijk HP et al., 2023 aimed to evaluate, in an in vivo clinical study on healthy human volunteers, the effects of sulforaphane from broccoli sprouts on the inflammatory, endothelial, and metabolic response induced by a caloric challenge, and the results showed a facilitation of a mild inflammatory response (increase in CCL-2 and low-grade inflammation score), without significant changes in metabolic stress markers [15]. Also, the results of Yanaka A et al., 2024 indicated that dietary approach with sulforaphane-rich broccoli sprouts decreases fecal calprotectin levels in patients with ulcerative colitis [16]. Possible neuroprotective and cognitive function-supporting effects are also reported, by reducing neuroinflammation and protecting the blood–brain barrier [17,18,19].

On the other hand, the development and consumption of sprouts also pose certain technological and food safety challenges. Due to the cultivation conditions (high humidity, moderate temperatures, high nutrient availability), sprouts are susceptible to microbial contamination and rapid spoilage, requiring strict hygiene, disinfection and packaging protocols, as well as gentle processing methods (drying, refrigeration, modified atmosphere packaging) to maintain nutritional quality and reduce the risks of foodborne illness [7,10].

Furthermore, the seed germination process triggers the activation of internal phytose enzymes, which hydrolyze phytic acid (phytate), thus reducing the concentration of this antinutrient and increasing the bioavailability of divalent minerals (Fe, Zn, Ca) [20,21].

A recent review notes that sprouting reduces the amounts of different anti-nutrients in the seeds and reduction in phytic acid has been found more profound than other compounds [9,22]. This suggests that sprouting can be adopted as a relatively simple technological strategy to improve the nutritional quality of cereals, pseudocereals and legumes by “deactivating” phytic acid.

In this context, a thorough understanding of the relationship between germination conditions, chemical composition changes (nutrients, bioactive compounds and antinutritional factors) and functional properties of sprouts is needed, as well as their potential to be integrated into innovative food products.

The aim of this study was to evaluate germination as a sustainable green pre-treatment for the recovery and enhancement of high-value compounds from broccoli and kale, by integrating nutritional and phytochemical characterization with antimicrobial assessment and structural analysis providing a scientific basis for the use of sprouts as functional ingredients in health-promoting food products.

The effect of germination on phytic acid is well documented for cereals, pseudocereals and legumes, where germination increases phytochemical activity and reduces phytate content, improving mineral bioavailability [20,21]. In cruciferous species, including broccoli and kale, available data regarding phytic acid changes during germination are limited and sometimes contradictory. While germination generally promotes phytate degradation through the activation of endogenous phytases [21,23], several studies have reported unchanged or even increased phytic acid levels in germinated *Brassica* seeds under specific conditions [24,25]. This variability may be explained by species- and cultivar-dependent differences in phytase activity, as well as by the strong influence of germination parameters such as time, temperature, moisture, and illumination [21]. Additionally, during the early stages of germination, phytic acid can be released from insoluble protein–mineral complexes, leading to a temporary increase in extractable phytate prior to its enzymatic hydrolysis [21].

Several studies have investigated the effects of germination on the nutritional and phytochemical composition of *Brassica* species, with a primary focus on specific bioactive compounds or antioxidant activity [13,14,15]. However, available literature on germinated broccoli and kale is often limited to targeted analyses and does not provide an integrated evaluation that simultaneously addresses nutritional composition, mineral bioaccessibility, anti-nutritional factors, antimicrobial functionality, and structural transformations. In this context, the present study contributes novel insights by combining compositional, functional, and structural analyses (FTIR and SAXS), thereby framing germination as a sustainable biological pre-treatment and green recovery strategy for high-value compounds. This comprehensive approach advances current knowledge and supports the valorization of broccoli and kale sprouts as functional ingredients within sustainable food systems.

## 2. Materials and Method

### 2.1. Seeds Germination

Two Cruciferous vegetables were analyzed: kale (*Brassica oleracea* var. *acephala*) and broccoli (*Brassica oleracea* var. *italica*). The seeds were obtained from BioSnacky Rapunzel, Sibiu, Romania. Prior to germination, the seeds were thoroughly washed and soaked in water for 12 h to facilitate and accelerate the germination process. Subsequently, they were placed on trays positioned near a natural light source, where germination occurred over a period of 2–3 days at a temperature range of 20–22 °C. Germination continued for another 5–6 days under the same temperature conditions until the sprouts formed [26]. During germination moisture content was monitored throughout the dehydration process. Germinated samples were dehydrated under controlled conditions, in an oven FloiLabo AC 60, Air Concept, Zülpich, Germany) at 50 °C for 3–4 days to achieve complete dehydration. The residual moisture was determined using a gravimetric method until to constant weight. This approach allowed confirmation of effective dehydration and minimized variability in subsequent nutritional, phytochemical, and structural analyses.

**Figure 1:**
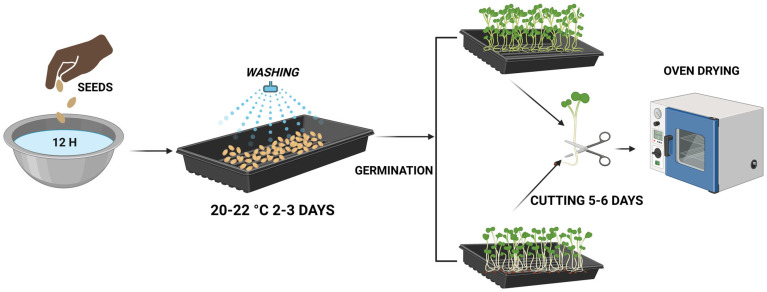
Technological flow for obtaining sprouts. Figure created with BioRender.com, accessed on 15 October 2025.

Figure 1 illustrates the technological flow of the seed germination and processing stages.

### 2.2. The Obtaining of Seed and Sprout Powders

**Figure 2:**
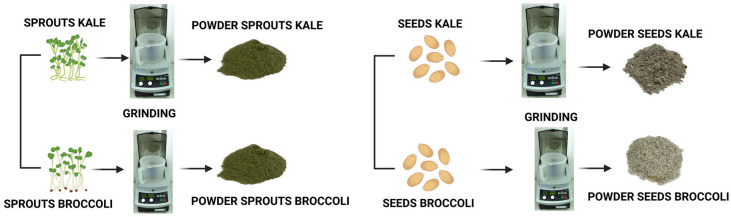
Technological flow for obtaining powders. Figure created with BioRender.com, accessed on 15 October 2025.

The dried sprouts and ungerminated seeds were ground using a kitchen blender (Bosch TSM6013B, BSH Hausgeräte, Munich, Germany) to obtain a fine, homogeneous powder while preserving nutrient integrity (Figure 2). The broccoli and kale samples were coded as is presented in Table 1.

### 2.3. Evaluation of the Nutritional Composition of Kale and Broccoli Seed and Sprout Powders

The nutritional composition of analyzed samples (protein, moisture, ash, carbohydrates and energy value) was determined using the methods presented in Table 2.

### 2.4. Phytochemical Analysis of Kale and Broccoli Seed and Sprout Powders

#### 2.4.1. Total and Individual Phenolics Content

To determine the total phenolic content (TPC), the Folin–Ciocalteu method was used [26]. This technique is based on the reduction in the Folin–Ciocalteu reagent by polyphenols leading to the formation of a blue complex that can be evaluated spectrophotometrically [30].

For analysis, the alcoholic extract was obtained by adding 10 mL of ethanol (70%) (Merck, Darmstadt, Germany) to 1 g of sample, mixing vigorously for 30 min in a shaker (IDL, Freising, Germany) [27]. After extraction, the solvent was removed under reduced pressure, and the extraction yield was calculated gravimetrically as the percentage of dry extract relative to the initial dry sample weight. TPC was determined using a calibration curve with a gallic acid standard (Merck, Darmstadt, Germany) in concentrations ranging from 2.50–200 µg/mL, and the results were reported in milligrams of gallic acid equivalents (GAE) per 100 g of sample. The correlation coefficient for the standard curve obtained was 0.9986, thus highlighting the reliability of the method [27,31]. Each analyzed sample was determined in triplicate. Chromatographic analysis of individual polyphenols was performed by HPLC according [26]. Caffeic acid, cumaric acid, rosmarinic acid, ferulic acid, beta-rezorcilic acid, epicatechin, resveratrol and quercitine were analyzed.

#### 2.4.2. Macro and Microelements Composition

For the determination of mineral content, the method described by Posta et al., 2022 was used [31]. Brefly, 3 g of the sample were subjected to incineration at 650 °C in a calcination oven (model L 9/11/B170, Nabertherm GmbH, Lilienthal/Bremen, Germany) until complete combustion of the organic matter was achieved. The resulting ash was then dissolved in 20% hydrochloric acid and filtered to obtain a clear solution, which was subsequently used for the quantification of macro- and microelements by atomic absorption spectroscopy (AAS).

Calibration curves were established using a certified multi-element standard solution (ICP Multi-Element Standard Solution IV, CertiPUR). The concentrations of the analyzed elements were expressed in parts per million (ppm). All determinations were conducted in triplicate to ensure analytical precision and result reproducibility. Nine macro and microelements were determined: Ca, Na, K, Mg, Cu, Zn, Fe, Mn and Ni.

### 2.5. Antinutritional Compounds (Phytic Acid) of Kale and Broccoli Seed and Sprout Powders

Phytic acid content was measured using the K-PHYT Phytic Acid/Total Phosphorus enzymatic-colorimetric kit (Megazyme/Neogen, Lansing, MI, USA), according to the most updated version of the supplier’s protocol (2025) [32]. The technique is based on the sequential enzymatic hydrolysis of phytic acid and lower inositol phosphates (IP_2_–IP_5_) to inorganic phosphate (Pi), followed by its colorimetric determination [33].

The sample (approx. 1.0 g) was trated with 0.66 M HCl, and the resulting supernatant after centrifugation was neutralized and subjected to the action of phytase for the degradation of phytic acid and alkaline phosphatase for the subsequent release of the inorganic ion. The released phosphate was evaluated using the molybdenum blue method, by measuring the absorbance at 655 nm [33,34].

### 2.6. Structural Analysis of Kale and Broccoli Seed and Sprout Powders

#### 2.6.1. Fourier Transform Infrared Spectroscopy (FTIR)

Prior to FTIR-ATR analysis, powders obtained from kale and broccoli seeds (KSeeds, BSeeds) and sprouts (KSprout, BSprout) were pre-dried at 40 °C overnight to remove residual moisture. FTIR spectra were collected according to the protocol described by Dossa et al. (2025) [34] using a Nicolet iS50 FT-IR spectrometer (Thermo Fisher Scientific, Waltham, MA, USA) equipped with an ATR accessory. Spectra were acquired over the 4000–500 cm^−1^ wavenumber range, averaging 32 scans at a spectral resolution of 4 cm^−1^. The obtained spectra were subsequently evaluated to identify characteristic functional groups and to compare the molecular fingerprints of kale and broccoli seed and sprout powders.

#### 2.6.2. Small- and Wide-Angle X-Ray Scattering (SAXS/WAXS)

SAXS and WAXS measurements were performed on powders obtained from kale and broccoli seeds (KSeeds, BSeeds) and sprouts (KSprout, BSprout), following the procedure described by Fluerasu et al. (2025) [35]. Analyses were carried out using a Xeuss 3.0 system (Xenocs SAS, Grenoble, France) equipped with a Cu Genix 3D X-ray source and a Dectris Eiger2 Si 1M detector (Dectris, Baden-Daettwil, Switzerland). Measurements were conducted under vacuum at room temperature using Kapton film. The sample-to-detector distances were set to 1800 mm for SAXS and 45 mm for WAXS, with H-Flux collimation and an acquisition time of 300 s. Data were processed using XSACT software (version 2.10; Xenocs, Grenoble, France). SAXS fitting was performed in the Guinier region (0.01–0.1 Å^−1^), assuming spherical particle geometry [35].

### 2.7. Determination of Antibacterial Activity of Kale and Broccoli Seed and Sprout Powders

The antibacterial activity of four powders was evaluated on eight nosocomial bacterial strains, of which four were Gram-negative and four were Gram-positive. The bacterial strains and the antibiotics used as positive control for each bacteria are presented in the Table 3. The tested strains were collected from patients who were hospitalized in different surgical departments, underwent surgical interventions, and then were detected with infections, at the time of presentation in the specialized outpatient clinic at the Misca Medical Center, Timișoara, Timiș County, Romania, where biological samples were collected and were inoculated on specific culture media in the microbiology laboratory of the Mișca Medical Center. All reference strains used are listed in Table 3 and stored in a freezer.

Isolation and confirmation of coliforms were carried out on selective culture media—MacConkey agar (Mikrobologie-Labor-Technik RO, Arad, Romania), Chromatic Detection Agar (Mikrobologie-Labor-Technik RO), and Eosine Methylene Blue (EMB–Levine)—and on confirmation media—Triple Sugar Iron (TSI) Agar, Simmons Citrate Agar, and Mobility Indole Urease (MIU) agar (Mikrobologie-Labor-Technik RO)—for *E. coli* and *K. pneumoniae*, respectively. The isolation of the *P. aeruginosa* germ was carried out on the Columbia agar culture medium with 5% ram red blood cells (Mikrobologie-Labor-Technik RO) and on the differential medium Chromogenic agar UTI (CHROMagar^TM^, Saint-Denis, France), on which the production of a characteristic green pigment is highlighted. The identification of *A. baumanii* used Leeds Acinetobacter Medium (Hardy Diagnostics, Santa Maria, CA, USA) and the differential medium Chromogenic agar UTI (CHROMagar^TM^) for cultivation, and thermostating was carried out for 24 h at 35 °C.

For the isolation and confirmation of the *S. aureus* germ, Chapman agar (Mannitol Salt agar) and Columbia agar with 5% red blood cells (Mikrobologie-Labor-Technik RO) are used, the isolation and confirmation of the *E. faecalis* germ was carried out on the selective culture media Chromatic Detection Agar (Mikrobologie-Labor-Technik RO) and Bile Aesculin Azide Agar (Mikrobologie-Labor-Technik RO). Columbia agar with 5% red blood cells (Mikrobiologie-Labor-Technik RO) was used for *S. pyogenes* and the confirmation was done by the sensitivity test to bacitracin.

For the isolation and identification of *S. pneumoniae*, Columbia agar with 5% red blood cells (Mikrobiologie-Labor-Technik RO) was used and the differentiation was done by the optochin test. The seeding is carried out in Petri plates with a diameter of 100 mm, by streaking with the help of sterile microbiological loops, in aseptic conditions, and the resulting plates are incubated for 24 h at 40–42 °C, for coliforms and at 35–37 °C, for staphylococcus, faecal enterococcus and streptococci. The methods used to evaluate the antibacterial activity were performed according to the European Committee on Antimicrobial Susceptibility Testing (EUCAST) [36] and with minor modifications based on our previous studies [37].

#### 2.7.1. Kirby-Bauer Disk Diffusion Susceptibility Test Protocol

For antimicrobial experiment, seed and sprout powders, prepared as is presented in Section 2.2. were used. at a dilution in water of 10^−1^. This dilution was used for the Kirby-Bauer Disk Diffusion Susceptibility method and for the CIM evaluation, the tested concentrations and the results are presented in the tables.

In order to quickly verify the effectiveness of thesprout and seed powders, on the eight microbial strains, the Kirby-Bauer technique, the diffusometric method, taking the antibiotic of choice as a standard for each of the bacterial strains was used. Thus, it was prepared the microbial suspension at an optical density (OD) of 0.5 McFarland, read at a wavelength of 565 nm, using standard saline for each tested strain and plated it on Mueller-Hinton agar (Sigma-Aldrich, Taufkirchen, Germany). After drying the inoculum, on the surface of the culture medium, the microtablet with the antibiotic of choice, for the positive control, was placed on sterile Whatman filter paper no. 1, which was soaked with 2 µL of the extracts to be tested. It should be noted that the diameter of the microtablets is 6 mm. After placing the microtablets on the surface of the culture medium, the plates are incubated for 18–24 h, at 35–37 °C, after which the size of the inhibition zones is checked by measuring the halo around the microtablets and expressing the results in millimeters [36].

#### 2.7.2. Determination of the Minimum Inhibitory Concentration (MIC)

Using the serial dilution method, the microbial suspension was adjusted to OD 5 × 10^5^ CFU/mL (colony forming units) in Mueller Hinton broth at a wavelength of 565 nm read on a McFarland densitometer. Thus, with each of the tested strains, the inoculum was made, at a concentration of 5 × 10^5^ CFU/mL and then 100 µL was taken and distributed in 5 standard test cups, sterile, each, over which 2, 4, 6 and 10 µL of each of the four test powders was added at a dilution of 10^−1^. For each microorganism tested, the experiments are performed in triplicate.

After 24 h of incubation at 37 °C, the development of microorganisms in each of the cups was checked, compared to the control. Wells in which a cloudy suspension or sediment appeared were considered positive. The minimum inhibitory concentration (MIC) was considered the lowest concentration of antimicrobial agent, which completely inhibits the development of microorganisms, in the microdilution cups. The working technique used for MIC determination is standardized by EUCAST to which we made minimal changes from our previous studies [36].

### 2.8. Statistical Analysis

Results are presented as mean values ± standard deviation (SD). Differences between means were assessed using Tukey HSD, JASP 0.95.4 followed by a multiple comparison analysis performed with a *t*-test in Microsoft Excel 365. Statistical significance was set at *p* < 0.05. All determinations were performed in triplicate.

## 3. Results and Discussion

### 3.1. Nutritional Composition of Kale and Broccoli Seed and Sprout Powders

The nutritional composition of broccoli (BSeeds, BSprouts) and kale (KSeeds, KSprouts) powders showed clear species- and stage-dependent differences (Table 4).

Germination markedly modified the proximate profile of both Brassica species. Sprout powders exhibited slightly lower moisture (1.83–2.30%) compared with seed powders (2.58–2.76%). According to Kowitcharoen et al. (2021), microgreens possess a more porous and hygroscopic structure after germination, which can slightly modify moisture behavior depending on dehydration conditions [38].

Ash content followed a species-dependent trend. In broccoli, ash increased from 0.55% (seeds) to 2.41% (sprouts), indicating enhanced mineral availability caused by phytate degradation and mineral release during sprouting—a mechanism also highlighted by Weber et al. (2017) in their mineral profiling of Brassica microgreens [39].

Conversely, kale showed a reduction in ash from 1.43% to 0.93%, consistent with genotype-specific mineral redistribution patterns previously reported within Brassica oleracea microgreens [39].

Protein content increased markedly during germination. Broccoli protein rose from 10.32% to 30.33%, while kale increased from 11.79% to 30.21%. This ~3-fold increase is consistent with the protein range commonly reported for microgreens (25–35 g/100 g DW), as summarized in the comprehensive review by Bhaswant et al. (2023) [40].

Lipid content decreased dramatically in both species. Broccoli lipids dropped from 30.91% to 2.65%, while those for kale varied from 30.90% to 3.45%. This observation is fully aligned with the biochemical role of triacylglycerols as the primary energy source during early sprout development, where β-oxidation rapidly consumes stored oils.

Although complete proximate profiles for Brassica sprout powders are limited in the literature, similar lipid reductions (60–80%) have been documented in sprouted vegetables and microgreens across multiple species [2,3]. Bhatt et al. (2023) also reported significant lipid depletion during drying and metabolic remobilization in broccoli microgreen powders [41].

Overall, germination significantly enhanced the nutritional quality of both broccoli and kale flours. Protein content tripled, lipids were reduced by over 85%, carbohydrates increased, and ash demonstrated species-specific behavior. These results are consistent with the physiological patterns reported in recent microgreen and sprout literature [38,39,40,41] and highlight the potential of Brassica sprout powders as functional, nutrient-dense ingredients suitable for fortified foods and plant-based formulations.

### 3.2. Total Phenolic Content and Individual Polyphenols of Kale and Broccoli Seed and Sprout Powders

**Figure 3:**
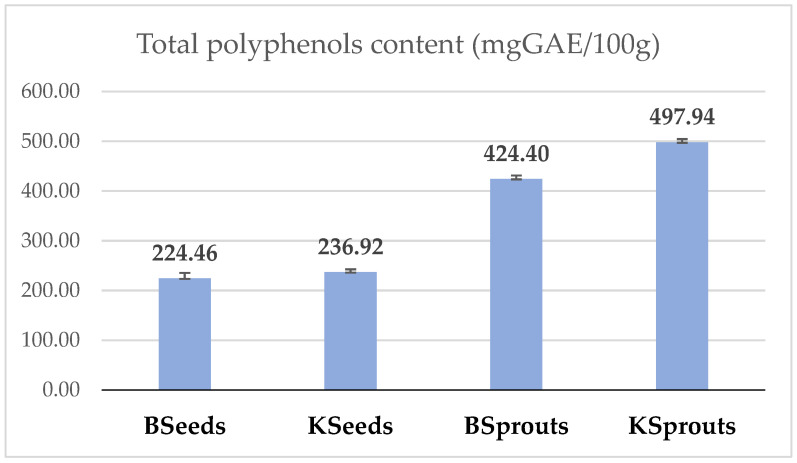
Total phenolic content (mgGAE/100 g) for BSeeds, KSeeds, BSprouts, Ksprouts. The values are expressed as mean values ± standard deviations of all measurements.

In Figure 3 is presented the total phenolic content (TPC) of broccoli and kale seeds and sprouts.

In terms of total phenolic content (mgGAE/100 g), BSprouts and KSprouts have a significantly higher value (424.40 ± 9.1 mg/100 g and 497.94 ± 4.5 mg/100 g) compared to BSeeds and KSeeds (224.46 ± 15.0 mg/100 g and 236.92 ± 5.1 mg/100 g). Thus, germination increased significantly total phenolic content by 52.42% for kale and by 47.11% for broccoli. Dziki et al., 2020 reported in broccoli sprouts an increase on total phenolics between 36.84–93.42% relative to the seeds, depending on drying, grinding processes and time of germination [42].

Broccoli seeds exhibited a TPC of 224.46 mg GAE/100 g, while kale seeds showed a slightly higher content (236.92 mg GAE/100 g), consistent with reports indicating that kale seeds generally possess a richer phenolic profile compared to broccoli due to inherent varietal differences in secondary metabolism [43].

The absolute values obtained for both broccoli and kale sprouts fall within the ranges previously reported for Brassica species, typically 350–800 mg GAE/100 g for broccoli sprouts and 350–900 mg GAE/100 g for kale sprouts.

Overall, the results demonstrate that germination is an effective strategy to enhance the phenolic content of Brassica materials, with kale showing the most pronounced increase. This suggests that kale sprouts may offer superior functional potential and antioxidant properties compared to broccoli sprouts, supporting their use as nutrient-rich ingredients in functional food formulations.

The extraction yields ranged from 7–10% for ungerminated seeds and 11–17% for germinated samples, with slightly higher values for broccoli compared to kale. The quantitative analysis of individual phenolic compounds in broccoli (BSprouts) and kale (KSprouts) sprout powders revealed substantial qualitative and quantitative differences between the two Brassicaceae species (Table 5). The major phenolic acids identified were caffeic, p-coumaric, ferulic, and rosmarinic acids, together with the flavonoids quercetin and resveratrol.

Broccoli sprout powder exhibited a remarkably high concentration of rosmarinic acid (200.87 µg/g DW)**,** followed by ferulic acid (25.40 µg/g DW). The predominance of rosmarinic acid suggests a strong antioxidant potential, exceeding the levels previously reported for fresh broccoli sprouts, which generally range between 40 and 90 µg/g DW [44,45]. Such an increase may result from the drying process, which enhances the release of bound phenolics from the plant matrix.

The presence of p-coumaric acid exclusively in broccoli sprouts (16.87 µg/g DW) indicates a species-dependent activation of the phenylpropanoid pathway. This observation aligns with findings by Pérez-Balibrea et al. in 2011 [46], who noted that p-coumarate derivatives are particularly abundant in later germination stages of broccoli.

In contrast, kale sprout powder showed a distinct profile dominated by caffeic acid (75.33 µg/g DW), in significantly higher concentrations than in broccoli. This pattern is consistent with reports by Baenas et al., 2014 [45], which demonstrated that kale germination enhances the synthesis of hydroxybenzoic and hydroxycinnamic acids through activation of key enzymes such as phenylalanine ammonia-lyase and polyphenol oxidase.

The amount of ferulic acid was comparable in both species (25.40 µg/g in BSprouts and 23.09 µg/g in KSprouts), confirming its relative stability throughout germination and dehydration processes. Similar levels were observed in other *Brassicaceae* sprouts such as mustard and rapeseed [45], suggesting a conserved phenolic pattern within this family.

A noteworthy difference was the detection of resveratrol (7.459 µg/g DW) only in kale sprouts. The occurrence of this stilbene may be linked to stress-induced metabolic responses during germination. Quercetin was found in both matrices (12.94 µg/g DW in broccoli and 9.40 µg/g DW in kale), values that correspond well with those reported by Martínez-Sánchez et al., 2008 [47] for broccoli sprouts (10–15 µg/g DW). The presence of this flavonol supports the antioxidant and anti-inflammatory potential of both samples.

Ungerminated broccoli and kale seeds exhibit a phenolic profile dominated by structural phenolic acids, such as ferulic and p-coumaric acid, while germination causes a significant increase in soluble phenolic compounds, including caffeic, and rosmarinic acid, as a result of activation of the phenylpropanoid pathway [44].

The values obtained for ungerminated broccoli and kale seeds fall within the ranges reported by Pająk, P. et al., 2014 [44] for Brassicaceae, confirming that the seed stage is characterized by a simple phenolic profile, dominated by phenolic acids. The quantitative differences observed, especially for caffeic acid, can be attributed to genetic variability and cultivation conditions, as also mentioned in the literature [44].

Overall, the broccoli sprout powder was characterized by a phenolic profile dominated by rosmarinic acid, while the kale sprout powder contained higher levels of caffeic and resveratrol. These differences reflect distinct biosynthetic pathways and enzymatic activities inherent to each species. The results confirm the high nutritional and functional value of both sprouts, reinforcing the role of Brassicaceae as natural sources of bioactive phenolics with antioxidant and health-promoting properties.

### 3.3. Macro and Microelements Composition of Kale and Broccoli Seed and Sprout Powders

The analysis of micro- and macroelements (Cu, Zn, Mn, Fe, Ni, Ca, Mg, K, Na) revealed significant differences between seeds and sprouts of the two *Brassica* species. The results indicate that the germination process selectively mobilizes minerals, influencing their accumulation depending on the species (Table 6).

In broccoli sprouts (BSprouts), marked increases were observed for Zn (4.27 to 7.30 mg/kg) and Mn (24.58 to 34.26 mg/kg). These values are slightly higher than the concentrations reported by Dobrowolska-Iwanek et al. (2022) [8], for broccoli sprouts (Cu: 4.9 mg/kg, Zn: 53.2 mg/kg, Mn: 20.2 mg/kg), confirming that sprouting enhances microelement mobility but also highlighting genotype- and methodology-related variation. In contrast, Fe exhibited only a minor increase (from 62.86 to 65.93 mg/kg), remaining below the value reported in the literature (143 mg/kg), most likely due to the dry-powder matrix used in the present study, which generally yields lower extractable Fe than fresh sprouts. Likewise, Kim et al. (2024) [48], observed similar increases in Cu and Zn concentrations in illite-treated broccoli sprouts, associating these minerals with improved antioxidant enzyme activity.

For kale sprouts (KSprouts), a higher Zn content (54.72 mg/kg) was recorded compared to the seeds (48.24 mg/kg), while both Fe content and Mn decreased during germination (Fe from 81.20 to 47.95 mg/kg, Mn from 13.61 to 10.42 mg/kg). These reductions can be attributed to mineral redistribution between cotyledons and elongating hypocotyl tissues, as well as potential leaching losses during seed imbibition, mechanisms previously described for *Brassica rapa* and *B. oleracea* sprouts [49].

Compared with recent literature, the mineral concentrations obtained in this study for broccoli sprouts are within the upper range of values reported for *Brassica* sprouts. For instance, Zn concentrations of 70.37 mg/kg and Cu levels of 7.30 mg/kg observed here are slightly higher than the ranges typically reported for broccoli sprouts in previous studies, where Zn generally varies between 40–60 mg/kg and Cu between 3–6 mg/kg on a dry weight bas is [8]. The moderately higher values found in the present study may be attributed to the longer germination period (7 days vs. 4–5 days in most literature reports), which allows extended mineral mobilization and enhanced enzymatic activity during sprout development [8].

The macroelements profile also demonstrated clear species-dependent differences. In broccoli, sprouting induced moderate increases in Ca (from 391.15 to 413.93 mg/kg) and Mg (from 60.14 to 62.59 mg/kg) relative to the seeds. Although the absolute values are lower than those reported by Dobrowolska-Iwanek et al. (2022) [8], who found Ca and Mg concentrations of 4130 mg/kg and 2950 mg/kg in fresh broccoli sprouts, the upward trend observed here is consistent with their findings. The discrepancies in magnitude can be attributed to methodological differences, as the present study analyzed dried sprout powders, whereas Dobrowolska-Iwanek et al. expressed mineral content on a fresh weight basis, which naturally yields higher mineral concentrations [8].

For kale sprouts, a substantial increase in K was observed (from 58.57 to 103.96 mg/kg), while Ca (from 348.37 to 329.06 mg/kg) and Mg (from 60.16 to 61.21 mg/kg) remained relatively stable or showed slight decreases compared with the seeds. This behavior corresponds with the variability reported by Tan et al. (2023), who demonstrated that Ca and K contents in kale microgreens are strongly dependent on species-specific growth characteristics and environmental conditions such as light spectrum and germination settings [49].

When compared to the ranges reported by Ebert (2022) [6] for Brassica microgreens (Ca: 850–1300 mg/kg, K: 280–360 mg/kg, Mg: 150–180 mg/kg on a dry matter basis), the values obtained in this study fall below these intervals. This difference is expected, given that the analyzed materials represent sprout powders, which undergo an additional dehydration step that alters the mineral distribution and reduces apparent concentrations compared with intact microgreens.

The elevated concentrations of Zn (70.37 mg/kg), Cu (7.30 mg/kg), and Mn (34.26 mg/kg) in broccoli sprout powder have important functional implications, as these trace elements serve as essential cofactors for antioxidant enzymes, including superoxide dismutase (SOD) and catalase. Similar associations between mineral enrichment and enhanced antioxidant capacity were reported by Kim et al. (2024), who demonstrated that increased levels of Zn and Cu during sprouting contribute to the activation of antioxidative metabolic pathways [48].

Overall, the results indicate that broccoli sprouts exhibit a more balanced and mineral-rich profile than kale sprouts, particularly regarding trace elements such as Zn, Cu, Mn, and Fe. The interspecific differences observed here can be attributed to genotypic variability, seed morphology, and species-specific metabolic processes activated during germination. These findings align with recent literature [6,8,48,49,50] and reinforce the potential of Brassica sprouts as valuable functional ingredients for mineral-enriched bakery products and plant-based formulations.

### 3.4. Antinutritional Compounds—Phytic Acid of Kale and Broccoli Seed and Sprout Powders

**Figure 4:**
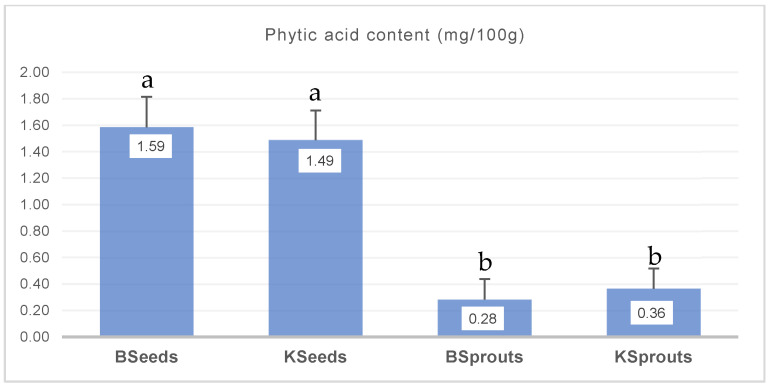
Phytic acid (mg/100 g) for BSeeds, KSeeds, BSprouts, Ksprouts. The values are expressed as mean values ± standard deviations of all measurements; data within the same column sharing different letters are significantly different (*p* < 0.05).

Figure 4 presents the phytic acid content of BSeeds, KSeeds, BSprouts and KSprouts.

The obtained results showed that broccoli and kale seeds, and broccoli and kale sprouts did not differ significantly from one another (*p* > 0.05). However, sprout powder exhibited significantly lower phytic acid content than seeds samples (*p* < 0.05). The reduction in phytic acid content after germination, compared to the values obtained in seeds, was 82.20% for broccoli and 75.47% for kale.

Phytic acid is recognized as the primary storage form of phosphorus in seeds and a major antinutrient due to its ability to chelate minerals such as iron, zinc, calcium and magnesium [6,51]. Numerous studies have shown that germination activates endogenous phytases, leading to enzymatic hydrolysis of phytate and subsequent reduction in phytic acid levels [6,52]. Our results show that both seed samples (BSeeds and KSeeds) exhibited higher phytic acid levels compared with their sprouted counterparts, confirming that sprouting reduces antinutrient content. This aligns with previous studies reporting that Brassica microgreens and sprouts undergo intensive metabolic remodeling during germination, enhancing enzymatic breakdown of storage compounds [6,52].

Overall, the present study confirms that germination is an effective natural method for reducing phytic acid, thereby improving mineral bioavailability and nutritional quality of Brassica-based ingredients. These results support the incorporation of sprouted powders as functional fortifiers in bakery formulations [6,52].

### 3.5. Structural Characteristics of Kale and Broccoli Seed and Sprout Powders

#### 3.5.1. FTIR Spectra

**Figure 5:**
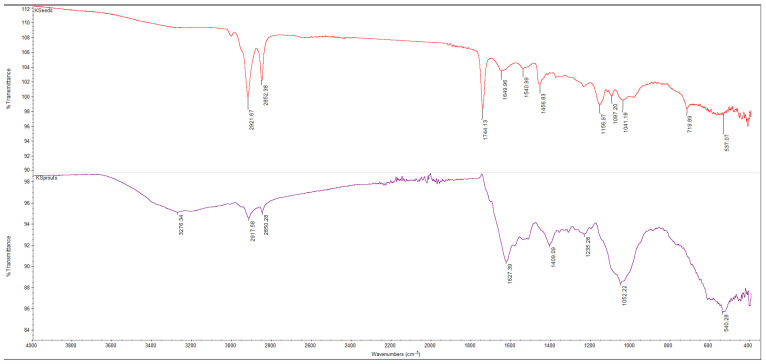
The FTIR spectra of the KSeeds (red line) and KSprouts (mauve line), spectral range of 4000–400 cm^−1^, 32 scans at 4 cm^−1^ resolution.

**Figure 6:**
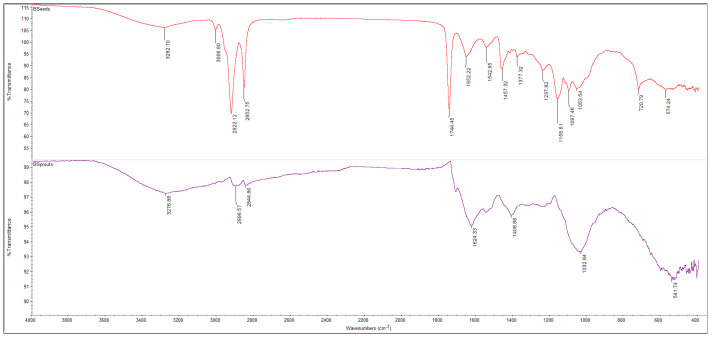
The FTIR spectra of the BSeeds (red line) and BSprouts (mauve line) spectral range of 4000–400 cm^−1^, 32 scans at 4 cm^−1^ resolution.

**Figure 7:**
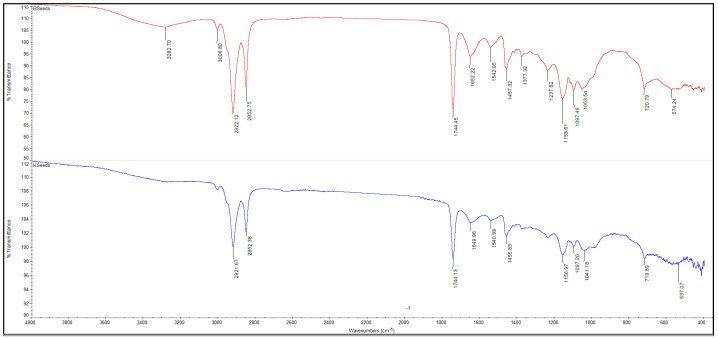
The FTIR spectra of the BSeeds (red line) and KSeeds (blue line), spectral range of 4000–400 cm^−1^, 32 scans at 4 cm^−1^ resolution.

**Figure 8:**
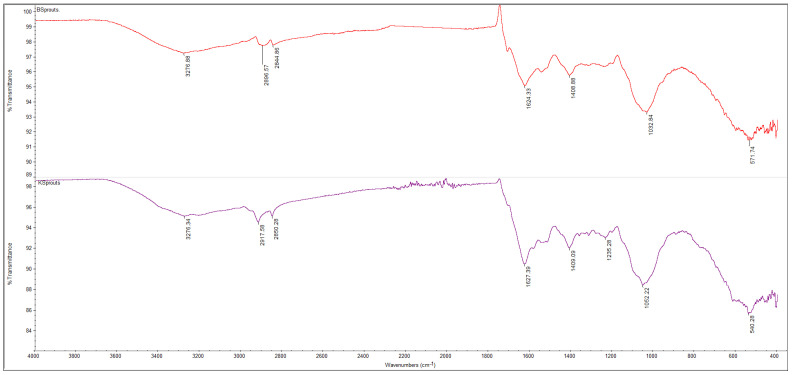
The FTIR spectra of the BSprouts (red line) and KSprouts (mauve line), spectral range of 4000–400 cm^−1^, 32 scans at 4 cm^−1^ resolution.

The FTIR spectra of powders obtained from broccoli and kale seeds, in both ungerminated (KSeeds, BSeeds) and germinated (KSprout, BSprout) forms, are presented in Figure 5, Figure 6, Figure 7 and Figure 8. The FTIR spectral profiles revealed marked differences in the chemical composition between seed and sprout powders. In comparison with the ungerminated samples, the powders derived from germinated seeds exhibited significant variations in both the intensity and position of the absorption bands within the investigated spectral range (4000–500 cm^−1^). These variations suggest structural and functional modifications of the main biochemical compounds, including proteins, lipids, and carbohydrates.

As demonstrated in Figure 5, the FTIR spectra of KSeeds and KSprouts highlight significant structural changes that are induced by the process of germination. In the KSeeds spectrum, the absorption bands at 2921 and 2852 cm^−1^ correspond to the stretching vibrations of methylene groups (–CH_2_/–CH_3_), which are characteristic of aliphatic chains in the fatty acids present in triglycerides and phospholipids [53,54,55,56]. The absorption band at 1744 cm^−1^, attributed to the C=O stretching vibrations of ester groups, is widely recognized as a hallmark of triglycerides and other lipid compounds. This spectral region is frequently employed in FTIR studies for the identification of lipids and the estimation of the esterified fraction of samples [53,54,55]. In the spectrum of KSprouts, there was a decrease in the intensity of the lipid-related bands. This suggests that there has been triglyceride hydrolysis and mobilization of fatty acids during metabolic processes, this phenomenon has been previously reported in other oilseeds [57]. The presence of a weak band at around 3276 cm^−1^ is attributed to the overlap of N–H (Amide A) and hydrogen-bonded O–H stretching vibrations, reflecting increased hydration and protein activity during germination [58]. The 1650–1540 cm^−1^ region, corresponding to amide I (C=O, C–N) and amide II (N–H, C–N) bands, exhibited changes indicative of protein reorganization and enzymatic activation, while intensified signals in the 1100–1000 cm^−1^ region reflected starch hydrolysis and an increase in soluble carbohydrate content, processes characteristic of seed germination [54,56,57].

Figure 6 presents the FTIR spectra of broccoli seed (BSeeds) and sprouts (BSprouts) samples. The broad signal observed around at 3282 cm^−1^ is attributed to the overlapping stretching vibrations of N–H (Amide A) and hydrogen-bonded O–H groups, reflecting the presence of proteins and weakly bound water molecules within the seed matrix [59,60]. In the BSeeds spectrum, the pronounced absorption bands at 2922 and 2852 cm^−1^ correspond to the stretching vibrations of methylene groups (–CH_2_/–CH_3_) in the aliphatic chains of fatty acids from triglycerides and phospholipids [55,56]. The distinct peak recorded at 1744 cm^−1^, indicative of C=O stretching vibrations from lipid esters, further confirms the presence of esterified lipids in the BSeeds sample. Additionally, the bands observed within the 1652–1457 cm^−1^ region are associated with amide I and amide II vibrations, characteristic of protein structures, while the absorptions in the 1237–1053 cm^−1^ region correspond to C–O–C and C–O stretching of polysaccharides and carbohydrates [60]. In the BSprouts spectrum, a marked decrease in the intensity of lipid-related bands (2900–2840 cm^−1^) indicates triglyceride hydrolysis and the utilization of fatty acids as an energy source during germination. The weak band at 3276 cm^−1^ suggests increased hydration and enhanced protein activity, corresponding to N–H/O–H stretching in proteins and bound water [59,60]. The region between 1624–1408 cm^−1^ becomes more pronounced, reflecting protein reorganization and enzymatic activation, while the intensified signals in the 1100–1000 cm^−1^ region are attributed to polysaccharide hydrolysis and the formation of soluble sugars (glucose, maltose), typical of the germination process [55,60].

The comparison of the FTIR spectra of broccoli (BSeeds) and kale (KSeeds) seeds (Figure 7) reveals a similarity in spectral profile, typical of the lipidic and protein compounds present in oilseeds. However, intensity differences are also observed, which reflect the specific compositional features of the two species. The two samples under scrutiny both exhibited characteristic lipid absorption bands at 2920–2850 cm^−1^ (–CH_2_/–CH_3_ stretching vibrations) and 1744 cm^−1^ (C=O stretching vibrations of ester groups). These bands were found to be slightly more intense in KSeeds, suggesting a higher lipid content. Conversely, the broad band observed around 3282 cm^−1^ in BSeeds indicates a greater contribution of N–H/O–H stretching vibrations, associated with proteins and hydroxyl-containing compounds, implying a relatively higher protein content. As can be seen in Figure 7, the regions 1650–1450 cm^−1^ (amide I and II) and 1200–1000 cm^−1^ (C–O–C, C–O stretching) are common to both spectra. However, minor variations in intensity are observed, attributable to differences in the relative proportions of proteins and carbohydrates [57,60].

The FTIR spectra of broccoli (BSprouts) and kale (KSprouts) sprouts (Figure 8) display similar spectral bands that are characteristic of the biochemical processes associated with germination. In addition, intensity variations can be observed that reflect compositional differences between the two species. The two samples under scrutiny both display absorption bands around 3276 cm^−1^, attributed to the stretching vibrations of N-H (Amide A) and hydrogen-bonded O-H groups. These groups are associated with increased hydration and enhanced protein activity during germination [59,60]. The bands within the 2900–2840 cm^−1^ region (–CH2, –CH3) are found to be weak, thus confirming the consumption of lipid reserves. The 1620–1400 cm^−1^ region, corresponding to amide I and II bands, is more intense in KSprouts, suggesting a higher protein content, while in BSprouts, the dominant signals appear in the 1100–1000 cm^−1^ range, associated with C–O–C and C–O vibrations of polysaccharides and soluble sugars, indicating a more pronounced mobilization of carbohydrates [58,59].

A comparative analysis of the FTIR spectra indicates significant structural and functional changes occurring during the germination of both broccoli and kale seeds. It has been demonstrated that the process of germination leads to the hydrolysis of triglycerides and a reduction in characteristic lipid bands (2920–2850 and 1744 cm^−1^), accompanied by an intensification of protein- and polysaccharide-related vibrations (1650–1400 and 1100–1000 cm^−1^); this indicates the mobilization of energy reserves and the activation of enzymatic metabolism. The enhancement of absorption signals around 3270–3280 cm^−1^ is indicative of increased hydration and the formation of hydrogen bonds associated with partially hydrolyzed proteins and carbohydrates. This phenomenon is associated with the onset of biosynthetic and cellular reorganization processes. The disparities in band intensities observed between the samples indicate species-specific metabolic behavior. Specifically, KSprouts manifest a more accentuated protein-related profile, while BSprouts demonstrate heightened carbohydrate and hydroxyl-associated activity. It is evident that these structural transformations indicate that germination signifies a pivotal stage in the reactivation of macro-molecules and the enhancement of the nutritional value of cruciferous seeds [54,55,56,57,58,59].

#### 3.5.2. Small-Wide Angle X-Ray Scattering (SAXS/WAXS)

**Figure 9:**
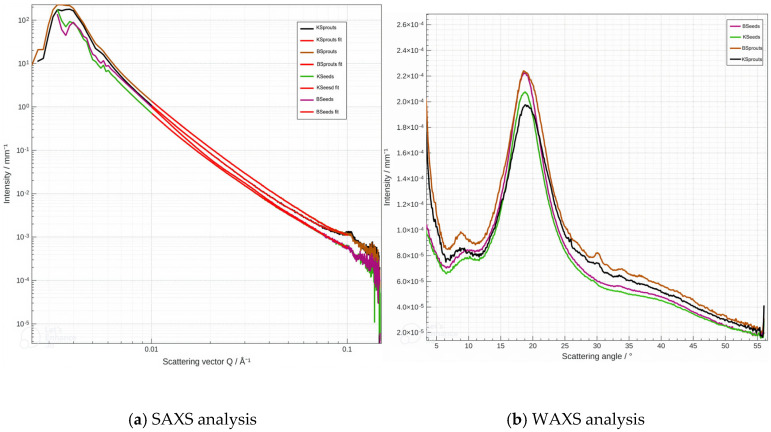
The analysis of Small-Wide Angle X-ray Scattering (SAXS/WAXS), BSeeds (pink line), KSeeds (light green line), BSprouts (orange line), KSprouts (black line).

SAXS and WAXS analyses of powders obtained from broccoli and kale seeds and sprouts (Figure 9a,b) revealed germination-induced modifications in nano-scale structural organization. The use of SAXS for characterizing food matrices enables the investigation of variations in size, shape, and electron-density contrast within nano-organized domains, particularly in protein, polysaccharide, and lipid structures [61,62]. All samples exhibited a Guinier-type behavior in the low-q region (0.01–0.1 Å^−1^). Differences in intensity and slope between seed and sprout samples indicate variations in effective domain size or electron-density contrast, reflecting structural reorganization during germination. The WAXS diffractograms showed a predominantly amorphous component together with narrow crystalline contributions, consistent with the semicrystalline nature of starch and other carbohydrate-based biopolymers [63]. Variations in peak shape and intensity between seeds and sprouts suggest changes in the partially crystalline fractions as structural reserves are mobilized during germination. Overall, the SAXS and WAXS results demonstrate that germination induces not only compositional changes but also internal structural reorganization of the powders, in agreement with observations reported for food biopolymers undergoing biochemical transformations [61,62].

The SAXS profiles obtained for the powders derived from broccoli and kale seeds and sprouts (Figure 9a) show a slight decrease in intensity with increasing scattering vector qqq, a trend that is characteristic of complex food matrices rich in biopolymers. It is evident that all samples manifest a distinct Guinier-type behavior within the low-q region (0.01–0.1 Å^−1^), thereby signifying the existence of nanometrically dispersed domains, a phenomenon that has been frequently documented in the context of food biopolymers [62]. The lower intensities and steeper slopes that were observed for the seed powders (KSeeds, BSeeds) in comparison with their sprouted counterparts (KSprouts, BSprouts) suggest that germination leads to an increase in the effective size or electron-density contrast of these domains. Such changes are consistent with the redistribution and mobilization of structural reserves (i.e., starch, proteins and lipids). This process has been well documented in studies on germinated cereals and legumes [64]. The SAXS profiles of KSeeds and BSeeds demonstrate a high degree of similarity, thus indicating that the nano-organization in dry seed powders is comparable. However, the more pronounced differences observed between KSprouts and BSprouts suggest that germination induces structural modifications that are specific to the species. As Atudorei et al. (2021) [60] have previously demonstrated, analogous observations have been reported in which germination leads to structural and compositional transformations that vary across species. The analysis of the SAXS parameters (Figure 9a and Table 7) further highlights clear differences between seed and sprout powders. BSeeds exhibited the smallest characteristic dimensions of the nano-organized domains (median = 819.84 nm; surface-equivalent mean size = 29.69 nm), whereas KSeeds displayed slightly larger structures (median = 841.82 nm; 38.86 nm). The process of germination served to accentuate the disparities observed, with BSprouts exhibiting augmented values (median = 849.29 nm; 43.08 nm) and KSprouts demonstrating the most substantial dimensions amongst all the samples (median = 865.21 nm; 53.03 nm). This progression is indicative of an expansion of the nano-organized domains following germination, a phenomenon that is more pronounced in kale than in broccoli.

The WAXS diffractograms (Figure 9b) exhibit a broad amorphous halo in the 2θ = 17–20° region, superimposed with narrow crystalline reflections around 15°, 17–18° and 23°, a pattern characteristic of semicrystalline plant biopolymers such as starch [63]. The position of the main peak remains nearly unchanged across samples, whereas the intensity of the reflections varies, indicating differences in the relative proportions of amorphous and crystalline phases. The higher intensities observed in the sprout samples suggest a structural reorganization of carbohydrate domains during germination, a phenomenon also reported for germinated cereals and legumes. BSprouts show the highest crystalline peak intensities, followed by BSeeds and KSprouts, while KSeeds display the lowest. These differences are consistent with the structural changes associated with the mobilization and reorganization of carbohydrate reserves throughout germination. The relative increase in crystalline peak intensity in sprouts compared with seeds aligns with processes of structural reordering and partial remodeling of starch and other polysaccharides during germination, when carbohydrate reserves are mobilized and granular structure is reorganized [64]. When considered as a whole, the SAXS and WAXS results demonstrate that the process of germination instigates discernible structural transformations in broccoli and kale seed powders. SAXS reveals an enlargement of nano-organized domains, a phenomenon that is more pronounced in sprout samples. In contrast, WAXS confirms changes in the balance between amorphous and crystalline carbohydrate phases. The present findings demonstrate that the process of germination exerts a significant influence on the molecular composition and the nano- to micro-scale architecture of powders, resulting in substantial alterations to their physicochemical properties. The combined evidence confirms that germination leads to extensive molecular and structural reorganization, thereby underscoring its substantial impact on the functional characteristics of plant-derived powders.

### 3.6. Antibacterial Activity of Kale and Broccoli Seed and Sprout Powders

**Figure 10:**
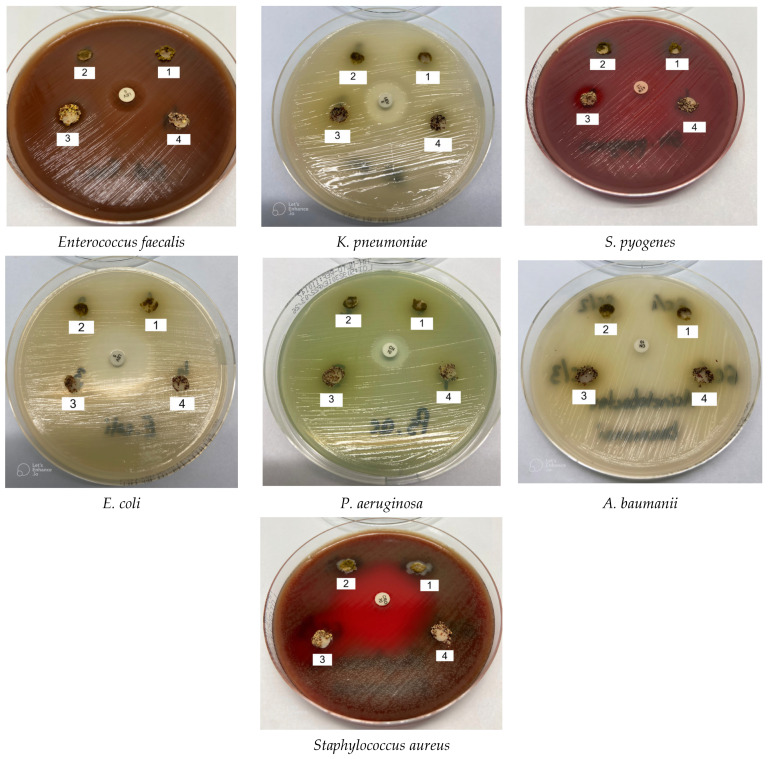
The antimicrobial effect of 4—BSeeds, 3—KSeeds, 2—BSprouts, 1—KSprouts against *E. coli*, *Enterococcus faecalis*, *K. pneumoniae*, *S. pyogenes*, *P. aeruginosa*, *A. baumanii*, *Staphylococcus aureus*.

The in vitro antibacterial activity of the four analyzed powders, at a dilution of 10^−1^, on the eight bacterial strains was evaluated qualitatively and quantitatively by the presence or absence of inhibition zones, in the case of the diffusometric method, as well as by the MIC values (Figure 10).

In Table 8 the zones of inhibition are highlighted—the diameter of the halo where the susceptible interruption points are around the antibiotic microtablets, which are considered as a control, compared to that of the tested powders.

It should be emphasized that the diameters of the inhibition zone, for each positive control and for each extract, include the 6 mm diameter of the micro-tablet compared to the tested microorganisms. All strains on which the effectiveness of the powders obtained is verified belong to nosocomial germs.

Analyzing the appearance of the halos on each of the Petri dishes, it was found that among the analyzed powders, only KSeeds proved its effectiveness against *E. coli* among the Gram-negative bacteria tested, and against the other tested germs the powders remained without effect. Evaluating the effect of these powders against the tested Gram-positive germs—*S. aureus*, *E. faecalis*, *S. pyogenes* and *S. pneumoniae*—it was observed that the KSeeds powder exerts a bactericidal effect on all four bacteria, BSeeds only inhibited the development of *E. faecalis* and *S. pyogenes* bacteria, being without effect on *S. aureus* and *S. pneumoniae* respectively. Regarding the KSprouts and BSprouts powders, respectively, they proved ineffective against all four tested Gram-positive strains.

For the *Escherichia coli* strain, it was found the effectiveness of the antibiotic of choice used—the microtablet with 5 µg of levofloxacin, which generated an inhibition zone—halo of 30 mm. Dilution of the tested powders has no effect for this strain of *E. coli*, the colonies developing to the vicinity of the microtablets soaked with 2 µL of the suspension each, except for the KSeeds powder, which generates a 12 mm diameter halo. All three other Gram-negative strains—*K. pneumoniae*, *P. aeruginosa* and *A. baumanii* are resistant to these analyzed powders.

For *K. pneumoniae*, the antibiotic considered positive control—the microtablet with 5 µg of levofloxacin, generates a halo of 17 mm, for *P. aeruginosa*, 10 µg of gentamicin, generates a halo of 15 mm, and for *A. baumanii*, the antibiotic considered positive control—the microtablet with 10 µg of gentamicin, has no effect on this strain. For *S. aureus*, the halo for microtablet with 30 µg of linezolid, considered a positive control, was of 31 mm.

Of the four powders tested at a dilution of 10^−1^ KSprouts, BSprouts, BSeeds are ineffective against the tested strain of *Staphylococcus aureus* at a volume of 2 µL of dilution, but KSeeds generated a halo of 16 mm.

For *E. faecalis*, the microtablet with 5 µg of levofloxacin, considered a positive control, generated a halo of 16 mm. Analyzing the effect of the powder KSeeds, at the dilution of 10^−1^, at a volume of 2 µL of dilution, generated a halo of 14 mm, while the sample BSeeds, under the same conditions, generated a halo of 12 mm, the other extracts checked, KSprouts and BSprouts, being without effect for this strain.

In the case of the *S. pyogenes* germ, analyzed, the microtablet with 10 µg of azithromycin, considered a positive control, has no effect on this germ, the colonies developing up to the proximity of the microtablet. Analyzing the effect of the powder KSeeds, at the dilution of 10^−1^, at a volume of 2 µL of dilution, it generated a halo of 14 mm, while the sample BSeeds, under the same conditions, generated a halo of 12 mm, the other tested powders—KSprouts and BSprouts, being without effect for this strain. The analysis of the effect of the obtained powders on the *S. pneumoniae* germ, referring to the microtablet with 30 µg of ceftriaxone, considered a positive control that is very effective against this strain, generating a halo with a diameter of 43 mm, we find that the effect of the KSeeds powder, at the dilution of 10^−1^, at a volume of 2 µL of dilution, generated a halo of 20 mm, while the rest of the samples checked—KSprouts and BSprouts and BSeeds—remained without effect for this strain. The diameters of the halos represent, in all cases, the arithmetic averages of the 3 samples.

Analyzing the tables presented in the document issued by EUCAST, regarding the size of the halos that indicate the antibiotic sensitivity of various microorganisms, we will find that in the majority, antibiotic substances that generate halos larger than 14 mm are considered effective, intermediately sensitive those between 12–14 mm, and resistant, those that generate halos smaller than 12 mm (in the case of the *Fam. Enterobacteriaceae*). Thus, we can state that *E. coli* is intermediately sensitive to the KSeeds powder.

In the case of the *Staphylococcus aureus* species, if we take linezolid as a standard, for a halo size greater than 24 mm, the strain is sensitive (S), between 21–24 mm, it is considered intermediate sensitive (IS), and below 21 mm, it is resistant (R), according to EUCAST. Considering other antibiotics that generate inhibition zones between 12 and 18 mm, and in our case, the strain of *Staphylococcus aureus* on which the Kseeds powder was tested and that generates a halo of 16 mm, at a dilution of 10^−1^ and a volume of 2 µL, can be considered sensitive.

Analyzing the behavior of the *Enterococcus faecalis* species, if we take levofloxacin as a standard, for a halo size greater than 22 mm, the strain is sensitive (S), between 19–22 mm, it is considered intermediate sensitive (IS), and below 19 mm, it is resistant (R), according to EUCAST. There are antibiotics that generate zones of inhibition up to 11 mm that are considered in the range. The strain of *Enterococcus faecalis* on which the powders obtained by us were tested, which generates halos between 12 and 14 mm, we can consider as intermediately sensitive, at a volume of 2 µL for both powders—KSeeds and BSeeds respectively. Evaluating the behavior of the *Streptococcus pyogenes* germ, relative to azithromycin, for an inhibition zone size greater than 21 mm, the strain is sensitive (S), between 18–21 mm, it is considered intermediate sensitive (IS), and below 18 mm, it is resistant (R), according to EUCAST. Considering that the strain of *Streptococcus pyogenes* shows inhibition zones of 12 mm exposed to BSeeds and 14 mm exposed to KSeeds, it must be considered resistany to both powders, according to Eucast.

Studying the germ of *S. pneumoniae* and the size of the zone of inhibition reported to ceftriaxone greater than 35 mm, the strain is sensitive (S), between 32–35 mm, it is considered intermediate sensitive (IS), and below 32 mm, it is resistant (R), according to EUCAST. However, there are also antibiotics where the zone of inhibition is between 8 and 14 mm, in range, according to Eucast. Thus, referring to this value, we can consider that *S. pneumoniae* is sensitive to KSeeds, which generates a halo of 20 mm.

In Table 9, the concentration range for each of the tested powders is highlighted—between 200 µg/mL and 1000 µg/mL—and the MIC values for each of the antibiotics are tested as a control. The MIC values for the analyzed extracts, in relation to the tested microorganisms, are as follows:

KSprouts—has no effect on all microbial strains analyzed at all volumes tested;

BSprouts—without effect for all microbial strains analyzed, at all volumes tested.

For KSeeds powder, which was tested at a dilution of 10^−1^ and volumes of 2, 4, 6 and 10 µL/100 µL of inoculum, the MIC was found only at 10 µL/100 µL of inoculum, for *E. coli*, *S. pyogenes*, *S. pneumoniae*, *E. faecalis* and *S. aureus*. For all other suspension volumes of powders at 10^−1^ dilution, these microorganisms showed microbial growth. All other microbial strains are resistant to these powders.

For the powder BSeeds, which was tested at a dilution of 10^−1^ and volumes of 2, 4, 6 and 10 µL/100 µL of inoculum, the MIC was found only at 10 µL/100 µL of inoculum, for *S. pyogenes*, *E. faecalis* and *S. aureus*. In the case of the other powders, they were without effect for the microbial strains analyzed at all the dilution volumes analyzed.

Seeds are known to contain concentrated defense-related compounds, including antimicrobial peptides, phenolics, and glucosinolate-derived metabolites, which are mobilized or metabolized during germination. As germination progresses, these compounds may be diluted, transformed into non-antimicrobial forms, or redistributed toward growth-related metabolism, thereby reducing their effectiveness in solid powder-based antimicrobial assays. Consequently, while germination enhances nutritional quality and reduces antinutritional factors, it does not necessarily preserve or amplify antimicrobial activity, which remains strongly dependent on the plant matrix, compound stability, and extraction conditions.

The antimicrobial activity observed against the tested bacterial strains, including Gram-positive (e.g., *Staphylococcus aureus*) and Gram-negative bacteria (e.g., *Escherichia coli*), is consistent with previous reports on *Brassica* sprouts. Vale et al., 2015 demonstrated that broccoli and kale sprouts exhibit inhibitory effects against several foodborne pathogens, with Gram-positive bacteria generally showing higher sensitivity to sprout extracts [65]. In addition, broccoli sprouts have been reported to exert antibacterial activity through sulfur-containing compounds such as sulforaphane, which are particularly effective against Gram-negative bacteria, including *Helicobacter pylori* [66,67].

Broccoli crude extracts showed inhibitory effects against pathogenic bacteria (e.g., *Bacillus cereus*, *Staphylococcus aureus*) and yeasts, suggesting the presence of antimicrobial components (proteinaceous and other phytochemicals) [68].

Tamokou et al., 2017 provide an extensive overview of the antimicrobial activities of medicinal spices and vegetables, emphasizing the role of phenolic compounds, glucosinolates, and other secondary metabolites as key contributors to antimicrobial efficacy [69]. Although the chapter does not specifically focus on kale or broccoli sprouts, it reports antimicrobial effects associated with plant seeds and vegetative tissues rich in phenolics and sulfur-containing compounds, which are characteristic of *Brassica* species [69]. Therefore, the antimicrobial effects identified in this work are consistent with the broader antimicrobial patterns reported for plant-derived seeds and sprouts rich in bioactive phytochemicals.

In conclusion, we can state that *E. coli* and *Enterococcus faecalis* twins are intermediately sensitive to KSeeds powder, at a dilution of 10^−1^ and a volume of 2 µL, and strains of *Staphylococcus aureus* and *S. pneumoniae* are sensitive to KSeeds powder, at a dilution of 10^−1^ and a volume of 2 µL. *Enterococcus faecalis* is also intermediately sensitive to BSeeds powder, at a dilution of 10^−1^ and a volume of 2 µL. The *Streptococcus pyogenes* strain is considered resistant to both KSeeds and BSeeds powders, although it generates inhibition zones of 14 and 12 mm, respectively, but insufficient according to Eucast regulations. Otherwise, all analyzed strains are resistant to the analyzed powders—KSprouts and BSprouts.

## 4. Conclusions

This study provides an integrated and original assessment of germination as a sustainable biological pre-treatment for the recovery and enhancement of high-value compounds from broccoli and kale. By combining nutritional, phytochemical, antimicrobial, and advanced structural analyses (FTIR and SAXS), the paper demonstrates that sprouting significantly improves protein content, total phenolics and mineral content, while reducing phytic acid levels, concomitant with pronounced molecular and structural reorganization of the plant matrix. Antimicrobial screening against eight clinically relevant strains revealed a matrix-dependent response, with selective inhibitory effects confined mainly to seed powders, particularly kale seeds, whereas sprout powders were inactive under the tested conditions. These findings highlight that, while germination effectively enhances nutritional quality and lowers antinutritional burden through a green bio-recovery mechanism, antimicrobial activity is not necessarily amplified and depends strongly on the plant matrix and compound distribution. Overall, the study advances current knowledge by positioning Brassica sprouting as a sustainable strategy for producing nutrient-dense functional ingredients, rather than antimicrobial agents, thereby offering a nuanced perspective on the functional outcomes of germination.

Future studies should focus on optimizing the germination process, by controlling environmental parameters, to maximize the accumulation of bioactive compounds and evaluate whether the antimicrobial potential can be modulated by such interventions. In addition, the application of omics techniques and advanced structural analyses could provide a deeper understanding of the molecular reorganization induced by germination. Finally, investigation of the technological applications of germ powders in real food matrices, along with safety assessments, will support their development as value-added functional ingredients.

## Figures and Tables

**Table 1:** The analyzed samples of Kale and Broccoli Seed and Sprout.

Samples	Scientific Name	Samples Code
Broccoli seeds	(*Brassica oleracea* var. *italica*)	BSeeds
Kale seeds	(*Brassica oleracea* var. *acephala*)	KSeeds
Broccoli sprouts	(*Brassica oleracea* var. *italica*)	BSprouts
Kale sprouts	(*Brassica oleracea* var. *acephala*)	KSprouts

**Table 2:** The methods used for nutritional composition of kale and broccoli seeds and sprouts.

Parameters	Methods	References
Protein content (%)	Protein content was calculated based on the nitrogen content obtained by the Kjeldahl method, using a conversion factor of 6.5.	[27]
Moisture (%)	Moisture content was measured by drying the samples at 105 °C until a constant mass was obtained, then the results were expressed as a percentage of the total weight of the sample.	[27]
Ash (%)	Ash content was determined by incinerating the samples in a muffle furnace at 650 °C, until complete removal of organic materials.	[28]
Fat (%)	Fat content was determined using petroleum ether solvent with Soxhlet equipment (Velp Scientifica SER 148 Solvent Extractor, Via Stazione 16, USMATE, Italy).	[29]
Carbohydrates (g/100 g)	The carbohydrate content was calculated as fraction remaining in the sample after taking into account moisture, ash, fat and protein using the formula: carbohydrate (%) = 100 − (moisture + ash + fat + protein)	[27]
Energy value (kcal)	Energy value was calculated according to the formula: kcal = (Proteins × 4) + (Carbohydrates × 4) + (Fat × 9)	[27]

**Table 3:** The analyzed bacterial strains.

Bacterial Strain	Family	Order	Antibiotic (Positive Control)
Gram-negative			
*Escherichia coli (E. coli)*	Fam. Enterobacteriaceae,	Ord. Enterobacterales	levofloxacin
*Klebsiella pneumoniae (K. pneumoniae)*	Fam. Enterobacteriaceae	Ord. Enterobacterales	levofloxacin
*Pseudomonas aeruginosa (P. aeruginosa)*	Fam. Pseumonadaceae	Ord. Pseudomonadales	gentamicin
*Acinetobacter baumanii (A. baumanii)*	Fam. Moraxellaceae	Ord. Pseudomonadales	gentamicin
Gram-positive			
*Staphylococcus aureus (S. aureus)*	Fam. Staphylococcaceae	Ord. Bacillales	linezolid
*Enterococcus faecalis (E. faecalis)*	Fam. Enterococcaceae	Ord. Lactobacillales	levofloxacin
*Streptococcus pyogenes (S. pyogenes)*	Fam. Streptococcaceae	Ord. Lactobacillales	azitromicyn
*Streptococcus pneumoniae (S. pneumoniae)*	Fam. Streptococcaceae	Ord. Lactobacillales	ceftriaxone

**Table 4:** Proximate composition of Kale and Broccoli Seed and Sprout Powders.

Samples	Nutritional Characteristics					
	Moisture	Ash	Proteins	Lipids	Carbohydrates	Energy Values
	(%)	(%)	(%)	(%)	(g/100 g)	(Kcal/100 g)
seed powders						
BSeeds	2.58 ± 0.82 ^a^	0.55 ± 0.27 ^a^	10.32 ± 0.30 ^a^	30.91 ± 0.04 ^a^	55.64 ± 0.43 ^a^	542.07 ± 3.21 ^a^
KSeeds	2.76 ± 0.11 ^b^	1.43 ± 0.12 ^b^	11.79 ± 0.70 ^b^	30.90 ± 0.29 ^a^	53.12 ± 0.76 ^b^	537.72 ± 1.10 ^b^
sprout powders						
BSprouts	1.83 ± 0.41 ^c^	2.41 ± 0.15 ^c^	30.33 ± 0.66 ^c^	2.65 ± 0.22 ^b^	62.77 ± 0.90 ^c^	396.27 ± 2.20 ^c^
KSprouts	2.30 ± 0.37 ^d^	0.93 ± 0.22 ^d^	30.21 ± 0.17 ^c^	3.45 ± 0.19 ^c^	63.12 ± 0.42 ^c^	404.35 ± 2.33 ^d^

The values are expressed as mean values ± standard deviations of all measurements; data within the same column sharing different superscripts are significantly different (*p* < 0.05).

**Table 5:** The individual polyphenols content (µg/g DW).

Samples								
	Epicatechin	Caffeic Acid	Beta-Rezorcilic Acid	P-Coumaric Acid	Ferulic Acid	Rosmarinic Acid	Resveratrol	Quercetin
Composite powder								
BSprouts	ND	7.16 ± 1.2 ^a^	ND *	16.87 ± 0.6 ^a^	25.40 ± 0.5 ^a^	200.87 ± 1.3 ^a^	ND	12.94 ± 1.9 ^a^
KSprouts	ND	75.33 ± 0.7 ^b^	ND	ND	23.09 ± 1.5 ^a^	17.68 ± 2.6 ^b^	7.46 ± 2.3 ^a^	9.40 ± 1.2 ^b^
BSeeds	ND	3.45 ± 1.3 ^c^	ND	9.14 ± 0.2 ^b^	35.25 ± 0.5 ^b^	ND	ND	9.86 ± 1.5 ^b^
KSeeds	ND	35.34 ± 0.8 ^d^	ND	ND	76.02 ± 0.7 ^c^	ND	ND	8.31 ± 2.6 ^b^

The values are expressed as mean values ± standard deviations of all measurements; data within the same column sharing different superscripts are significantly different (*p* < 0.05); data within the same column sharing the same superscripts are not significantly different (*p* > 0.05). * ND-not detectable.

**Table 6:** The macro and microelements composition of sprouts and seeds (mg/kg).

Samples	Elements								
	Cu	Ni	Zn	Fe	Mn	Ca	Mg	K	Na
seed powders									
BSeeds	4.27 ± 0.02 ^a^	0.74 ± 0.06 ^a^	39.15 ± 0.29 ^a^	62.86 ± 0.15 ^a^	24.58 ± 0.23 ^a^	391.15 ± 8.90 ^a^	60.14 ± 0.23 ^a^	59.13 ± 0.09 ^a^	26.01 ± 0.05 ^a^
KSeeds	3.26 ± 0.06 ^b^	3.72 ± 0.34 ^b^	48.24 ± 0.57 ^b^	81.20 ± 1.08 ^b^	13.61 ± 0.24 ^b^	348.37 ± 13.39 ^b^	60.16 ± 0.04 ^a^	58.58 ± 0.65 ^a^	30.52 ± 0.16 ^b^
Sprout powders									
BSprouts	7.30 ± 0.13 ^c^	1.03 ± 0.06 ^c^	70.37 ± 1.32 ^c^	65.93 ± 0.46 ^c^	34.26 ± 0.14 ^c^	413.93 ± 8.39 ^c^	62.59. ± 0.16 ^a^	61.21 ± 0.17 ^a^	39.20 ± 0.18 ^c^
KSprouts	3.86 ± 0.09 ^ab^	2.03 ± 0.06 ^d^	54.72 ± 0.61 ^d^	47.95 ± 0.74 ^d^	10.42 ± 0.07 ^b^	329.06 ± 0.13 ^b^	61.21 ± 0.27 ^a^	103.96 ± 0.33 ^b^	37.88 ± 0.20 ^d^

The values are expressed as mean ± standard deviation; data within the same column sharing different superscripts are significantly different (*p* < 0.05).

**Table 7:** SAXS parameters of kale and broccoli seed and sprout powders.

Sample	Median of the Distribution, nm	Surface Equivalent Mean Size, nm	Goodness of Fit
BSeeds	819.84	29.69	1.27
KSeeds	841.82	38.86	1.04
BSprouts	849.29	43.08	1.00
KSprouts	865.21	53.03	1.01

**Table 8:** The evidence of the antibacterial activity of the tested powders, by the Kirby-Bauer Disk Diffusion Susceptibility method.

The Tested Microorganism	The Halo Diameter of the Antibiotic Used as a Control	The Halo Diameter of 2 µL Suspension Dilution 10^−1^ KSprouts	The Halo Diameter of 2 µL Suspension Dilution 10^−1^ BSprouts	The Halo Diameter of 2 µL Suspension Dilution 10^−1^ KSeeds	The Halo Diameter of 2 µL Suspension Dilution 10^−1^ BSeeds
*E. coli*	Levofloxacin 30 mm	6 mm	6 mm	12 mm	6 mm
*K. pneumoniae*	Levofloxacin 17 mm	6 mm	6 mm	6 mm	6 mm
*P. aeruginosa*	Gentamicin 15 mm	6 mm	6 mm	6 mm	6 mm
*A. baumanii*	Gentamicin 6 mm	6 mm	6 mm	6 mm	6 mm
*S. aureus*	Linezolid 31 mm	6 mm	6 mm	16 mm	6 mm
*E. faecalis*	Levofloxacin 16 mm	6 mm	6 mm	14 mm	12 mm
*S. pyogenes*	Azitromicyn 6 mm	6 mm	6 mm	14 mm	12 mm
*S. pneumoniae*	Ceftriaxone 43 mm	6 mm	6 mm	20 mm	6 mm

**Table 9:** Minimum inhibitory concentration (MIC) values for the different extracts as well as for the antibiotics used as reference standards.

	MIC µg/mL	KSprouts µg/mL				BSprouts µg/mL				KSeeds µg/mL				BSeeds µg/mL			
		200	400	600	1000	200	400	600	1000	200	400	600	1000	200	400	600	1000
*E. coli*	Levofloxacin 0.06	R	R	R	R	R	R	R	R	R	R	R	IS	R	R	R	R
*K. pneumoniae*	Levofloxacin 0.06	R	R	R	R	R	R	R	R	R	R	R	R	R	R	R	R
*P. aeruginosa*	Gentamicin 1	R	R	R	R	R	R	R	R	R	R	R	R	R	R	R	R
*A. baumanii*	Gentamicin 1	R	R	R	R	R	R	R	R	R	R	R	R	R	R	R	R
*S. aureus*	Linezolid 2	R	R	R	R	R	R	R	R	R	R	R	S	R	R	R	S
*E. faecalis*	Levofloxacin 1	R	R	R	R	R	R	R	R	R	R	R	IS	R	R	R	IS
*S. pyogenes*	Azitromicyn 1	R	R	R	R	R	R	R	R	R	R	R	IS	R	R	R	IS
*S. pneumoniae*	Ceftriaxone 0.1	R	R	R	R	R	R	R	R	R	R	R	S	R	R	R	R

R—resistant, IS—intermediate sensitive, S—sensitive.

## Data Availability

The original contributions presented in the study are included in the article; further inquiries can be directed to the corresponding authors.
